# Patterns of analgesic use to relieve tooth pain among residents in British Columbia, Canada

**DOI:** 10.1371/journal.pone.0176125

**Published:** 2017-05-01

**Authors:** Jamie Moeller, Julie Farmer, Carlos Quiñonez

**Affiliations:** Discipline of Dental Public Health, Faculty of Dentistry, University of Toronto, Toronto, Ontario, Canada; Virginia Commonwealth University, UNITED STATES

## Abstract

The use of prescription opioids has increased dramatically in Canada in recent decades. This rise in opioid prescriptions has been accompanied by increasing rates of opioid-related abuse and addiction, creating serious public health challenges in British Columbia (BC), one of Canada's most populated provinces. Our study explores the relationship between dental pain and prescription opioid use among residents in BC. We used data from the 2003 Canadian Community Health Survey (CCHS), which asked respondents about their use of specific analgesic medications, including opioids, and their history of tooth pain in the past month. We used logistic regression, controlling for potential confounding variables, to identify the predictive value of socioeconomic factors, oral health-related variables, and dental care utilization indicators. The Relative Index of Inequality (*RII*) was calculated to assess the magnitude of socioeconomic inequalities in the use of particular analgesics by incorporating income-derived ridit values into a binary logistic regression model. Our results showed that conventional non-opioid based analgesics (such as aspirin or Tylenol) and opioids were more likely to be used by those who had experienced a toothache in the past month than those who did not report experiencing a toothache. The use of non-opioid painkillers to relieve tooth pain was associated with more recent and more frequent dental visits, better self-reported oral health, and a greater income. Conversely, a lower household income was associated with a preference for opioid use to relieve tooth pain. The RII for recent opioid use and conventional painkiller use were 2.06 (95% CI: 1.75–2.37) and 0.62 (95% CI: 0.35–0.91), respectively, among those who experienced recent tooth pain, suggesting that adverse socioeconomic conditions may influence the need for opioid analgesics to relieve dental pain. We conclude that programs and policies targeted at improving the dental health of the poor may help to reduce the use of prescription opioids, thereby narrowing health inequalities within the broader society.

## Introduction

The use of prescription opioids has increased dramatically during the past two decades in Canada. While opioids have been used historically for symptomatic pain relief [1–2], the health care community in North America traditionally restricted their use to treating severe acute pain following trauma or surgery, and chronic pain related to terminal malignancies [3]. However, prescription opioids, most notably Oxycodone (Oxycontin™), emerged as a mainstream solution to address Canada’s untreated chronic pain problem in the mid-1990s and, by doing so, was expected to mitigate the social and economic costs to Canadian society [3–5]. Prescription opioids were proposed as an alternative to COX inhibitors, such as acetaminophen or ibuprofen, whose use and efficacy as analgesics were limited by their side-effects and toxicity [2]. This coincided with a growing consensus among health care professionals that opioids were an appropriate treatment to address other forms of chronic pain which were not cancer-related–suitably named chronic non-cancer pain (CNCP); these conditions included back pain, osteoarthritis, fibromyalgia, and headaches, among other disorders [3].

Yet, while their ability to offer relief for acute pain and cancer-related pain is undisputed and well-demonstrated through clinical studies [6–7], their use for treating CNCP is more controversial [8–9]. Opioids are associated with a wide variety of undesirable side effects, including constipation, drowsiness, and nausea or vomiting [10]. More concerning, however, is that opioids carry an inherent risk for abuse and addiction when administered long-term, and high doses of opioids–or opioids administered in conjunction with other respiratory depressants–can potentially be fatal [9]. Indeed, opioid-related deaths in several Canadian jurisdictions have grown in tandem with the prescription opioids dispensed in recent years [11–12], a trend closely mirrored by the United States during the past decade [13].

In fact, increasing rates of addiction, substance abuse, and opioid-related fatalities have now created a “public health emergency” in British Columbia (BC) [14], one of Canada’s most populated provinces, which is largely driven by unsafe sources of opioids in the street drug market [15]. Nevertheless, like other Canadian provinces, over-prescribing patterns of opioids by health professionals in BC during the past two decades has likely contributed, at least in part, to the current provincial crisis [16], and to the broader national prescription drug abuse “epidemic” in Canada [12, 17–19]. In recent years, several policy measures have been proposed to address the problem, including: the defunding of particular opioid analgesics from most provincial public drug plans, albeit with questionable results [12]; expanding access to addiction services and harm reduction programs [20]; and, the development of new guidelines and modifying educational curricula for health care professionals [21]. To be sure, there appears to be a broad consensus forming among policy experts and health care professionals that a more multi-faceted approach is needed to address the underlying problem [22–25]. In this regard, it is imperative for policy-makers to understand the social and economic determinants of prescription drug use in order to target public resources to those populations at the highest risk for opioid abuse.

Certainly, several socioeconomic indicators have been identified as strong predictors for fatal overdoses with opioids, including poor educational attainment [26], low income [27], and homelessness [28], suggesting that a socioeconomic gradient exists with regards to opioid abuse and addiction [29]. Notably, sustained and untreated sources of dental pain are disproportionately concentrated among many of these same socioeconomic groups in Canada [30], which may heighten the potential for extended or chronic use of opioid analgesics in order to manage pain in the absence of definitive treatment. To be sure, Canadians with a lower socioeconomic status are considerably more likely to forgo or delay dental treatment than those from a higher socioeconomic position [31], often reflecting both personal and social barriers to accessing care [30–32]. In this regard, identifying treatable sources of pain (i.e. dental-related pain) that can be addressed or prevented altogether through non-pharmaceutical approaches may also offer potential remedies to this public health challenge by lessening the need for prescription opioids.

Given this, our study attempts to determine the association between dental pain and prescription opioid use among residents in BC. The objectives of our study are twofold: first, to identify which socioeconomic indicators and oral health-related factors are associated with conventional analgesic and opioid analgesic use among those who report having recently experienced a toothache; and, second, to assess the magnitude of socio-economic factors as a source of inequalities among those who use different types of analgesic medications to alleviate tooth pain.

## Materials and methods

### Conceptual framework

**Fig 1:**
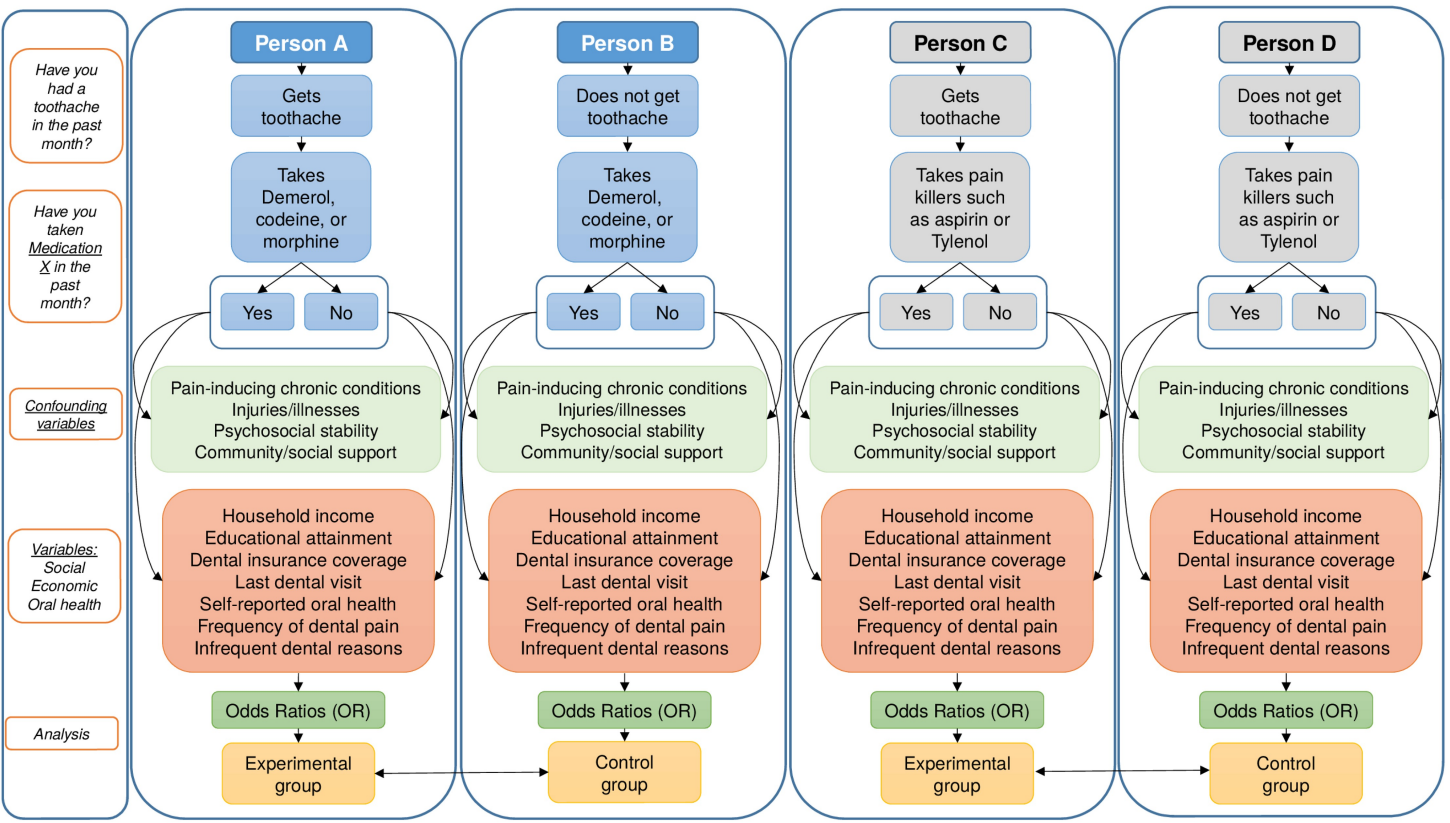
Conceptual framework illustrating a pathway in which Person A’s use of opioid analgesics may be affected by a toothache, and predicted by particular social, economic, and oral health factors. Person B’s use of opioids may be predicted by the same social, economic and oral health factors, but their use of opioids will act as a baseline by which to compare to Person A, because they did not experience a toothache. A similar pathway is repeated for Person C’s use of conventional painkillers such as Tylenol, using Person D as a comparative baseline.

Our conceptual framework uses a working hypothesis model [33] to approximate peoples’ potential to use different types of analgesics–namely, opioids, such as codeine or Demerol, and conventional pain relievers, such as aspirin or Tylenol–to relieve tooth pain. Linking peoples’ self-reported experience of tooth pain in their recent history and their self-reported use of particular analgesics in the same timeframe can establish an indirect relationship between analgesic medication patterns and tooth pain. Specifically, if we observe that particular subgroups of the population readily use certain analgesics in close proximity to self-reported tooth pain at a higher rate than their socioeconomic counterparts who have no history of tooth pain within the same time period, and after controlling for confounding variables (such as factors that affect pain tolerance, comorbidities, and age- and sex-specific factors that affect medication use), then we can indirectly examine use of analgesic medications and its socioeconomic context (*Fig 1*).

### Data source

We used data from the 2003 Canadian Community Health Survey (CCHS), a geographically-representative survey that collects demographic and socioeconomic information of respondents, as well as self-reported information pertaining to health status and health behaviours. Despite more recent surveys being available for use, the 2003 CCHS was chosen for its robustness of data for this analysis; namely, it is the most recent CCHS to ask respondents from BC to answer questions about both their self-reported tooth pain and their self-reported use of different analgesics within the past month. Statistically, the 2003 CCHS represents approximately 98 per cent of the BC population aged 12 years and older [34], although our analysis excluded those 19 years of age and younger. The 2003 CCHS data were weighted to reflect the age, sex, and location of the BC population. All data analyses were completed using Statistical Package for the Social Sciences (SPSS) Version 22.0 for Windows, unless otherwise noted.

### Variables and data analysis

This analysis focuses on two specific questions asked to participants: (1) recent experience of a toothache; and (2) recent use of analgesics within the same time frame. Respondents were asked “Have you had a toothache in the past month?” (yes or no) and “Have you used any of the following medications in the past month?” for which twenty-one possible options were given; our analysis focuses on only two classes of drugs: (1) painkillers such as aspirin or Tylenol (including arthritis medicine and anti-inflammatories); and (2) Demerol, codeine, or morphine (representing opioids). For these statements, respondents could answer either positively or negatively.

Demographic information was used, including: sex (male, female) and age (20–24 years, 25–34 years, 35–44 years, 45–54 years, 55–64 years, and 65 and older). Socioeconomic characteristics were used, including: educational attainment (less than high school, completion of high school, college education or other non-university post-secondary schooling, and completion of university degree), dental insurance coverage (yes or no) and total annual household income (less than $15,000, $15,000 - $29,999, $30,000 - $49,999, $50,000 - $79,999, and more than $80,000). As well, self-reported dental care utilization information was collected, including: last dental visit (less than one year, one to three years, three to five years, and more than five years) and estimated frequency of dental visits (more than once per year, once per year, less than once per year, and emergency care appointments only]. Self-reported oral health (excellent, very good, good, fair, and poor) and frequency of dental pain the past month (often, sometimes, rarely, never) were used as proxies for evaluating respondents’ oral health status.

Finally, potential chronic conditions and psychosocial comorbidities which may predispose respondents to concomitant use of analgesics were identified and incorporated into our multivariate regression model as confounding variables. Specifically, respondents were asked if they had several chronic conditions for which analgesics may be required, including: (i) fibromyalgia; (ii) arthritis or rheumatism, excluding fibromyalgia; and, (iii) back problems, excluding fibromyalgia and arthritis. Given that participants responded separately for each condition, a new variable was created which was positive for respondents who reported having one or more of the chronic conditions, and negative if respondents denied having any of the chronic conditions. As well, we controlled for self-reported injuries within the past 12 months (yes or no) and injuries due to repetitive strain within the past year (yes or no). Several indicators of psychosocial stability and mental health were also included: life satisfaction (satisfied or unsatisfied); self-reported mental health (excellent/very good/good, fair/poor); self-reported day-to-day amount of stress (none/not much/a bit, quite a lot/extremely); and, respondents’ sense of belonging to their local community (strong or weak).

We used a multi-stage process for our analysis. First, we divided respondents into two categories: those who reported experiencing a toothache in the past month, and those who reported not experiencing a toothache in the past month. Selecting only those respondents who experienced a toothache in the past month, we then used a multivariate regression model to determine if respondents had a greater likelihood to report taking: (i) opioid analgesics and (ii) painkillers such as aspirin or Tylenol based upon their socioeconomic and demographic indicators, dental care utilization patterns, and oral health status indicators described above. This process was then repeated for those respondents who did not report experiencing a toothache in the past month, thereby establishing a baseline against which each odds-ratio (β_x_) could be compared.

We then used the *Relative Index of Inequality* (*RII*) to determine the magnitude of socioeconomic inequalities within the study sample. The *RII* is a regression-based measure that takes into account the socioeconomic distribution of the population, thereby removing variability in the size of socioeconomic groups as a source of variation in the magnitude of inequalities in health [35–36]. The *RII* can be interpreted as the prevalence ratio of a particular outcome (i.e. opioid use) between people at the bottom and those at the top of a socioeconomic hierarchy [36]. The higher the *RII*, the more prevalent are socioeconomic inequalities within the study population, with a value of 1 indicating perfect equality between socioeconomic subgroups [29]. For instance, using household income as an indicator of socioeconomic position, an *RII* score of 2 would indicate that opioid use is twice as high among those with the lowest household income compared to those from the highest income households [37].

To calculate the *RII*, we first identified those variables which showed evidence of socioeconomic differences in outcomes (i.e. analgesic use). We then calculated *ridit* scores using Microsoft Excel™ and these values were incorporated into the binary regression models for each of the aforementioned models to determine respective *RII* measures, controlling for age, sex, and potential pain-related and psychosocial comorbidities of respondents [38]. The exponential of the regression coefficient was then taken as the *RII* [27] and percentage differences between the experimental and control groups’ *RII* values were calculated to assess the relative significance of these socioeconomic factors as a contributor to different patterns of analgesic use among those who experience tooth pain. Confidence intervals for the relative indices of inequality were produced using the standard error of the *ridit*-dependent exponential coefficient.

## Results

In total, 13,888 respondents answered questions pertaining to both their medication use within the past month and their history of a toothache during the same time period. Of those surveyed, 1,441 respondents (10.4%) reported having experienced a toothache within the past month, a finding which is consistent with other estimates of the prevalence of tooth pain among Canadians [30]. As well, 9,598 respondents (69.9%) reported having taken pain killers such as aspirin or Tylenol during that time, and 1,088 respondents (7.9%) reported receiving prescribed opioids such as Demerol, codeine, or morphine. Of those who experienced a toothache in the past month (*n* = 1,441), 1,119 respondents (77.7%) also reported having taken painkillers such as aspirin or Tylenol, and 213 respondents (14.8%) reported having taken prescribed opioids during the same timeframe.

The use of conventional painkillers such as aspirin or Tylenol was disproportionately concentrated among middle-aged adults aged 35–54 (71.7%), women (74.7%), and those reporting excellent oral health (71.6%). As well, use of these painkillers increased with income; those from the lowest income bracket reported the least frequent use (66.8%) while those from the highest income households reported the greatest use in the past month (72.7%). Those with non-university post-secondary education reported greater use of these painkillers (72.4%), as did those with dental insurance (72.8%). The use of Demerol, codeine, and morphine was likewise more often associated with middle aged adults (9.5%), women (9.1%), those with non-university post-secondary education (9.8%), and those with dental insurance (8.3%). Unlike conventional painkillers, however, opioids were prescribed more often for those with less income (10.3%) and those self-reporting poor oral health (14.0%). These findings are summarized in *Table 1*.

**Table 1:** Distribution of respondents, as well as proportion of respondents who report using Demerol, codeine, or morphine within the past month, using conventional painkillers such as Tylenol within the past month according to a variety of demographic, socioeconomic, and oral health indicators.

Characteristic		Distribution					
		General population (n = 13,888)		Reports using medication within past month:			
				*Demerol*, *codeine*, *or morphine (n = 1*,*088)*		*Painkillers such as aspirin or Tylenol (n = 9*,*598)*	
		Total	% (95% CI)	Total	% (95% CI)	Total	% (95% CI)
Age	20–24	738	5.4 [5.0–5.8]	65	8.8 [7.0–11.0]	500	67.8 [61.3–71.0]
	25–34	2,102	15.3 [14.7–15.9]	163	7.8 [6.7–9.0]	1,484	70.6 [68.6–72.5]
	35–44	2,593	18.9 [18.2–19.5]	240	9.3 [8.2–10.4]	1,869	72.1 [70.3–73.8]
	45–54	2,626	18.9 [18.5–19.8]	251	9.6 [8.5–10.7]	1,876	71.4 [69.7–73.1]
	55–64	2,195	16.0 [15.4–16.6]	167	7.6 [6.6–8.8]	1,490	67.9 [65.9–69.8]
	65 and older	3,486	25.4 [24.7–26.1]	202	5.8 [5.1–6.6]	2,379	68.2 [66.7–69.8]
Sex	Male	6,318	46.0 [45.2–46.8]	412	6.5 [5.9–7.2]	4,054	64.2 [63.0–65.3]
	Female	7,422	54.0 [53.2–54.9]	676	9.1 [8.5–9.8]	5,544	74.7 [73.7–75.7]
Household income	Less than $15,000	1,331	11.2 [10.7–11.8]	137	10.3 [8.8–12.0]	889	66.8 [64.2–69.3]
	$15,000 - $29,999	2,300	19.4 [18.7–20.1]	191	8.3 [7.2–9.5]	1,583	68.8 [66.9–70.7]
	$30,000 - $49,999	2,685	22.7 [21.9–23.4]	227	8.4 [7.5–9.6]	1,906	71.0 [69.2–72.7]
	$50,000 - $79,999	2,996	25.3 [24.5–26.1]	249	8.3 [7.4–9.4]	2,157	72.0 [70.4–73.6]
	$80,000 or more	2,534	21.4 [20.7–22.1]	177	7.0 [6.1–8.1]	1,841	72.7 [70.9–74.4]
Educational attainment	Less than high school	2,323	18.8 [18.2–19.5]	180	7.7 [6.7–8.9]	1,632	70.3 [68.4–72.1]
	High school graduate	2,782	20.7 [20.0–21.3]	183	6.6 [5.7–7.6]	1,884	67.7 [66.0–69.5]
	Non-university post-secondary	1,281	9.5 [9.0–10.1]	126	9.8 [8.3–11.6]	927	72.4 [69.9–74.8]
	Post-secondary graduate	7,096	52.6 [51.8–53.5]	583	8.2 [7.6–8.9]	4,992	70.3 [69.3–71.4]
Self-reported oral health	Excellent	2,666	19.9 [19.2–20.6]	201	7.5 [6.6–8.6]	1,910	71.6 [69.9–73.3]
	Very Good	4,052	30.2 [29.4–31.0]	272	6.7 [6.0–7.5]	2,808	69.3 [67.9–70.7]
	Good	4,325	32.2 [31.4–33.0]	346	8.0 [7.2–8.9]	2,997	69.3 [67.9–70.7]
	Fair	1,667	12.4 [11.9–13.0]	143	8.6 [7.3–10.0]	1,187	71.2 [68.9–73.3]
	Poor	708	5.3 [4.9–5.7]	99	14.0 [11.6–16.7]	494	69.8 [66.3–73.0]
Dental insurance coverage	Insured	7,561	56.8 [56.0–57.7]	628	8.3 [7.7–9.0]	5,502	72.8 [71.7–73.8]
	Non-insured	5,749	43.2 [42.4–44.0]	425	7.4 [6.7–8.1]	3,835	66.7 [65.5–67.9]

Among those reporting a toothache within the past month, more recent dental visits, more frequent dental visits, and dental insurance coverage were associated with a greater likelihood to require pain killers such as aspirin or Tylenol. While these trends also existed among those who reported experiencing a toothache in the past month, the differences widened; that is, the likelihood to use these pain killers increased for respondents who had a toothache in the past month. In contrast, differences in educational attainment appeared to be insignificant in determining the pattern of pain killer use. Those with less income were, however, considerably less likely to take these pain killers, and the divide between the highest and lowest income groups became greater among those with a recent toothache. Finally, those who self-reported better oral health were more likely to report using pain killers such as aspirin or Tylenol in the past month, regardless of experiencing a toothache. These patterns persisted after controlling for respondents’ age, sex, and comorbidities, although the differences became less pronounced. These findings are summarized in *Table 2*.

**Table 2:** Results of bivariate logistic regression analysis for the odds of using conventional painkillers such as aspirin or Tylenol among (1) those who do not report experiencing a toothache in the past month; and, (2) and those who report experiencing a toothache in the past month using a variety of demographic, socioeconomic, and oral health indicators.

Characteristic		Unadjusted OR*				Adjusted OR**			
		*Among those who report not having experienced a toothache in the past month*, *have you taken* pain relievers such as Tylenol in the past month*?*		*Among those who report having experienced a toothache in the past month*, *have you taken* pain relievers such as Tylenol in the past month*?*		*Among those who report not having experienced a toothache in the past month*, *have you taken* pain relievers such as Tylenol in the past month*?*		*Among those who report having experienced a toothache in the past month*, *have you taken* pain relievers such as Tylenol in the past month*?*	
		Odds Ratio (95% CI)	*P*	Odds Ratio (95% CI)	*P*	Odds Ratio (95% CI)	*P*	Odds Ratio (95% CI)	*P*
Age	20–24	0.92 [0.76–1.11]	0.358	1.14 [0.68–1.89]	0.626	1.19 [0.97–1.45]	0.091	1.10 [0.63–1.89]	0.745
	25–34	1.07 [0.94–1.21]	0.301	1.43 [0.91–2.24]	0.120	1.35 [1.18–1.55]	<0.001	1.37 [0.85–2.22]	0.196
	35–44	1.11 [0.99–1.23]	0.075	1.59 [1.03–2.46]	0.038	1.33 [1.17–1.51]	<0.001	1.50 [0.94–2.39]	0.089
	45–54	1.12 [0.99–1.25]	0.070	1.53 [0.97–2.41]	0.069	1.22 [1.08–1.39]	0.002	1.40 [0.86–2.26]	0.174
	55–64	0.97 [0.86–1.09]	0.603	1.22 [0.73–2.07]	0.450	1.04 [0.92–1.18]	0.554	1.07 [0.62–1.85]	0.809
	65 and older	Reference		Reference		Reference		Reference		
Sex^†^	Male	0.61 [0.57–0.66]	<0.001	0.61 [0.47–0.78]	<0.001	0.64 [0.59–0.69]	<0.001	0.61 [0.47–0.79]	<0.001
	Female	Reference		Reference		Reference		Reference		
Last reported dental visit^†^^‡^	Less than one year	1.05 [0.93–1.17]	0.430	1.83 [1.24–2.71]	0.003	1.10 [0.97–1.24]	0.156	1.56 [1.02–2.39]	0.041
	Between one and three years	1.00 [0.87–1.14]	0.961	1.26 [0.81–1.96]	0.315	1.07 [0.92–1.24]	0.393	1.30 [0.80–2.11]	0.298
	Between three and five years	0.87 [0.72–1.04]	0.123	1.13 [0.60–2.13]	0.701	0.90 [0.74–1.09]	0.275	1.33 [0.64–2.74]	0.437
	More than five years	Reference		Reference		Reference		Reference		
Educational attainment	Secondary education or less	0.83 [0.77–0.89]	<0.001	0.88 [0.70–1.11]	0.276	0.92 [0.84–1.00]	0.043	0.79 [0.60–1.04]	0.094
	Post-secondary education	Reference		Reference		Reference		Reference		
Household income^†^^‡^	Less than $15,000	0.76 [0.66–0.88]	<0.001	0.47 [0.29–0.75]	0.001	0.61 [0.52–0.73]	<0.001	0.36 [0.21–0.63]	<0.001
	$15,000 - $29,999	0.87 [0.76–0.98]	0.027	0.38 [0.25–0.59]	<0.001	0.73 [0.64–0.85]	<0.001	0.33 [0.20–0.54]	<0.001
	$30,000 - $49,999	0.92 [0.81–1.04]	0.194	0.72 [0.46–1.14]	0.166	0.86 [0.75–0.99]	0.029	0.58 [0.35–0.98]	0.044
	$50,000 - $79,999	0.98 [0.87–1.11]	0.773	0.54 [0.35–0.83]	0.005	0.97 [0.85–1.10]	0.617	0.47 [0.29–0.77]	0.003
	$80,000 or more	Reference		Reference		Reference		Reference		
Self-reported oral health^†^	Excellent	1.32 [1.08–1.62]	0.007	1.67 [1.06–2.89]	0.047	1.38 [1.10–1.74]	0.005	1.83 [0.89–3.78]	0.103
	Very Good	1.14 [0.93–1.39]	0.206	1.15 [0.77–1.71]	0.494	1.27 [1.02–1.58]	0.032	0.89 [0.57–1.40]	0.624
	Good	1.15 [0.95–1.41]	0.158	0.89 [0.62–1.28]	0.541	1.27 [1.02–1.58]	0.030	0.81 [0.81–1.23]	0.322
	Fair	1.20 [0.96–1.49]	0.110	0.83 [0.57–1.20]	0.318	1.31 [1.03–1.66]	0.029	0.77 [0.51–1.16]	0.216
	Poor	Reference		Reference		Reference		Reference		
Frequency of dental pain in past month^†^^‡^	Often	N/A		1.94 [1.30–2.89]	0.001	N/A		1.88 [1.17–3.01]	0.009
	Sometimes	N/A		1.65 [1.16–2.36]	0.005	N/A		1.74 [1.14–2.64]	0.010
	Rarely	N/A		1.25 [0.86–1.81]	0.242	N/A		1.10 [0.71–1.70]	0.683
	Never	N/A		Reference		N/A		Reference		
Dental services utilization frequency^†^	More than once per year	1.09 [1.00–1.20]	0.061	1.51 [1.14–2.00]	0.004	1.11 [1.00–1.23]	0.058	1.33 [0.96–1.85]	0.090
	Once per year	1.10 [1.00–1.22]	0.052	1.13 [1.13–2.05]	0.006	1.16 [1.04–1.30]	0.009	1.37 [0.97–1.93]	0.074
	Less than once per year	1.07 [0.92–1.24]	0.384	1.12 [0.72–1.76]	0.616	1.13 [0.96–1.33]	0.148	1.09 [0.66–1.81]	0.731
	Emergency only	Reference		Reference		Reference		Reference		
Dental insurance coverage^†^^‡^	Insured	1.30 [1.21–1.40]	<0.001	1.62 [1.28–2.05]	<0.001	1.36 [1.25–1.48]	<0.001	1.43 [1.10–1.86]	0.008
	Non-insured	Reference		Reference		Reference		Reference		

*Model 1: entered variables independently.

**Model 2: controlled for respondents’ age, sex, self-reported chronic conditions (fibromyalgia, arthritis, rheumatism, and/or back pain), life satisfaction, self-reported mental health, self-reported daily stress, sense of belonging to local community, self-reported injury within the past year, and self-reported injuries due to repetitive strain within the past year.

^**†**^ Statistically significant differences observed for this variable prior to adjusting for confounding variables at the 95% confidence level.

^**‡**^ Statistically significant difference observed for this variable after adjusting for potentially confounding variables at the 95% confidence level.

In contrast, indicators of dental care utilization appear to have less of an influence on determining patterns of opioid consumption (*Table 3*). That is, dental care utilization frequency and dental insurance did not predict the likelihood of having taken prescription opioids, such as Demerol, codeine, or morphine within the past month, irrespective of having experienced a toothache or not. Instead, self-reported oral health and socioeconomic factors appear to have a greater influence over patterns of prescription opioid use. Those who report poorer self-reported oral health were considerably more likely to have been prescribed opioid analgesics within the past month, although the general pattern and odds ratios are remarkably consistent among those who both report experiencing and not experiencing a toothache in the past month. On the other hand, those who reported having experienced tooth pain more frequently in the past month were significantly more likely to have taken prescription opioids compared to those who only sometimes or rarely experienced dental pain in the past month. Those with less educational attainment were considerably less likely to use prescription opioid analgesics than those with more education, and this difference became more pronounced among those who experienced a toothache; that is, those with more education were even more likely to have taken prescription opioids in the past month if they experienced a toothache. However, after controlling for age, sex, and comorbidities, these patterns disappear. With the exception of age, no independent variables showed statistically significant predictive properties for opioid use after controlling for confounding variables. Nevertheless, while statistically non-significant, there still appears to be a notable pattern of opioid prescriptions distributed according to respondents’ household income, albeit less pronounced than the pattern observed in our unadjusted model.

**Table 3:** Results of bivariate logistic regression analysis for the odds of using conventional painkillers such as aspirin or Tylenol among (1) those who do not report experiencing a toothache in the past month; and, (2) and those who report experiencing a toothache in the past month using a variety of demographic, socioeconomic, and oral health indicators.

Characteristic		Unadjusted OR*				Adjusted OR**		
		*Among those who report not having experienced a toothache in the past month*, *did you take* codeine, Demerol or morphine in the past month*?*		*Among those who report having experienced a toothache in the past month*, *did you take* codeine, Demerol or morphine in the past month*?*		*Among those who report not having experienced a toothache in the past month*, *did you take* codeine, Demerol or morphine in the past month*?*		*Among those who report having experienced a toothache in the past month*, *did you take* codeine, Demerol or morphine in the past month*?*
		Odds Ratio (95% CI)	*P*	Odds Ratio (95% CI)	*P*	Odds Ratio (95% CI)	*P*	Odds Ratio (95% CI)
Age*^†^	20–24	1.24 [0.87–1.78]	0.239	2.30 [1.11–3.77]	0.025	1.66 [1.14–2.42]	0.009	2.13 [0.99–3.58]
	25–34	1.18 [0.92–1.50]	0.187	2.03 [1.04–2.97]	0.037	1.51 [1.17–1.96]	0.002	1.94 [0.97–2.90]
	35–44	1.40 [1.13–1.75]	0.003	2.51 [1.32–3.78]	0.005	1.56 [1.23–1.98]	<0.001	2.27 [1.16–3.43
	45–54	1.61 [1.31–1.99]	<0.001	2.20 [1.13–3.28]	0.021	1.64 [1.32–2.06]	<0.001	1.84 [0.92–2.66]
	55–64	1.32 [1.05–1.65]	0.018	1.36 [0.61–2.01]	0.452	1.36 [1.07–1.72]	0.011	1.13 [0.50–2.55]
	65 and older	Reference		Reference		Reference		Reference	
Sex	Male	0.65 [0.56–0.75]	<0.001	0.89 [0.67–1.20]	0.456	0.68 [0.58–0.78]	<0.001	0.94 [0.69–1.28]
	Female	Reference		Reference		Reference		Reference	
Last reported dental visit	Less than one year	1.00 [0.81–1.24]	0.995	1.05 [0.62–1.80]	0.852	1.04 [0.82–1.30]	0.771	1.28 [0.73–2.24]
	Between one and three years	1.01 [0.78–1.30]	0.960	1.14 [0.63–2.08]	0.662	1.03 [0.79–1.35]	0.816	1.17 [0.63–2.19]
	Between three and five years	1.07 [0.76–1.51]	0.698	0.87 [0.36–2.12]	0.757	1.04 [0.72–1.49]	0.845	0.85 [0.34–2.17]
	More than five years	Reference		Reference		Reference		Reference	
Educational attainment^†^	Secondary education or less	0.73 [0.64–0.84]	<0.001	0.65 [0.48–0.86]	0.003	0.82 [0.70–0.96]	0.013	0.78 [0.56–1.09]
	Post-secondary education	Reference		Reference		Reference		Reference	
Household income^†^	Less than $15,000	1.35 [1.03–1.61]	0.043	1.97 [1.59–2.45]	0.008	1.12 [0.84–1.50]	0.426	1.37 [0.71–2.28]
	$15,000 - $29,999	1.14 [0.91–1.44]	0.258	1.36 [1.03–1.94]	0.038	1.02 [0.79–1.32]	0.859	1.18 [0.68–2.04]
	$30,000 - $49,999	1.07 [0.85–1.33]	0.572	1.51 [1.13–2.14]	0.027	1.07 [0.84–1.36]	0.590	1.21 [0.77–2.22]
	$50,000 - $79,999	1.16 [0.94–1.43]	0.175	0.93 [0.66–1.54]	0.778	1.20 [0.96–1.50]	0.119	0.94 [0.55–1.62]
	$80,000 or more	Reference		Reference		Reference		Reference	
Self-reported oral health^†^	Excellent	0.63 [0.46–0.88]	0.006	0.66 [0.38–0.97]	0.047	0.71 [0.50–1.02]	0.062	0.87 [0.54–1.57]
	Very Good	0.55 [0.40–0.76]	<0.001	0.50 [0.32–0.78]	0.002	0.64 [0.45–0.90]	0.011	0.70 [0.42–1.15]
	Good	0.68 [0.50–0.94]	0.018	0.46 [0.30–0.70]	<0.001	0.79 [0.57–1.11]	0.171	0.58 [0.36–0.92]
	Fair	0.55 [0.38–0.80]	0.002	0.74 [0.49–1.12]	0.154	0.58 [0.40–0.85]	0.006	0.92 [0.59–1.43]
	Poor	Reference		Reference		Reference		Reference	
Frequency of dental pain in past month^†^^‡^	Often	N/A		2.74 [1.57–4.76]	<0.001	N/A		1.94 [1.30–2.89]
	Sometimes	N/A		1.79 [1.04–3.07]	0.035	N/A		1.65 [1.16–2.36]
	Rarely	N/A		0.93 [0.51–1.71]	0.822	N/A		1.25 [0.86–1.81]
	Never	N/A		Reference		N/A		Reference	
Dental services utilization frequency	More than once per year	0.87 [0.73–1.03]	0.109	0.79 [0.56–1.10]	0.163	0.90 [0.74–1.08]	0.256	1.00 [0.68–1.46]
	Once per year	0.93 [0.77–1.11]	0.423	0.70 [0.49–1.02]	0.061	0.93 [0.77–1.14]	0.494	0.84 [0.56–1.26]
	Less than once per year	1.05 [0.80–1.37]	0.731	1.08 [0.64–1.82]	0.788	1.03 [0.78–1.37]	1.03	1.00 [0.57–1.79]
	Emergency only	Reference		Reference		Reference		Reference	
Dental insurance coverage	Insured	1.14 [0.99–1.31]	0.074	0.96 [0.72–1.27]	0.758	1.18 [1.01–1.37]	0.039	0.94 [0.69–1.27]
	Non-insured	Reference		Reference		Reference		Reference	

*Model 1: entered variables independently.

**Model 2: controlled for respondents’ age, sex, self-reported chronic conditions (fibromyalgia, arthritis, rheumatism, and/or back pain), life satisfaction, self-reported mental health, self-reported daily stress, sense of belonging to local community, self-reported injury within the past year, and self-reported injuries due to repetitive strain within the past year.

^**†**^ Statistically significant differences observed for this variable prior to adjusting for confounding variables at the 95% confidence level.

^**‡**^ Statistically significant difference observed for this variable after adjusting for potentially confounding variables at the 95% confidence level.

Nevertheless, the Relative Index of Inequality (*RII*) helps to illustrate the magnitude of any income-related differences for analgesic use in this sample. After controlling for respondents’ age, sex, and comorbidities, the *RII* for use of conventional analgesics (i.e. Tylenol) among respondents was 0.82 (95% CI: 0.73–0.91) for those who did not experience a toothache in the past month, and dropped to 0.63 (95% CI: 0.35–0.91) among those who reported experiencing a toothache in the past month–a difference of 23.2 per cent, albeit statistically non-significant. On the other hand, the *RII* for the need for prescription opioids such as Demerol, codeine, or morphine within the past month was 2.06 (95% CI: 1.75–2.37) and 1.02 (95% CI: 0.87–1.17) among those who did and did not experience a toothache in the past month, respectively. This difference was statistically significant, and suggests that the magnitude of income-related inequalities for opioid prescriptions is roughly twice as great among those who had experienced a toothache (*Table 4*).

**Table 4:** Results of imputed Relative Indices of Inequality (RII) for conventional painkiller and opioid use according to respondents’ reported household income, and the proportional changes predicted by the presence and absence of a toothache in the past month.

Medication class	Medication example	Relative Index of Inequality (RII)*		RII percentage difference
		*Report not having experienced a toothache within the past month*	*Report having experienced a toothache in the past month*	
Conventional non-opioid analgesic	Aspirin or Tylenol	0.82 [0.73–0.91]	0.63 [0.35–0.91]	-23.2%
Opioid analgesic	Demerol, codeine or morphine	1.02 [0.87–1.17]	2.06 [1.75–2.37]	+102.0%

*Model 1: controlled for respondents’ age, sex, self-reported chronic conditions (fibromyalgia, arthritis, rheumatism, and/or back pain), life satisfaction, self-reported mental health, self-reported daily stress, sense of belonging to local community, self-reported injury within the past year, and self-reported injuries due to repetitive strain within the past year.

## Discussion

In this study, we analyzed responses from a provincially-representative sample of British Columbians in 2003, which may not be entirely reflective of today’s population. However, it should be noted that the socioeconomic differences observed in our study are consistent with a more recent study examining prescription opioid use in BC [39]. In fact, these more recent findings indicate that socioeconomic divisions have become more pronounced during the past decade, suggesting that our findings and recommendations may be even more felicitous for policy-makers today.

We found that just over 10 per cent of those living in BC, one of Canada’s most populated provinces, experienced tooth pain in the past month. Of those, almost 80 per cent reported taking painkillers such as aspirin or Tylenol in the past month, compared to roughly 70 per cent of the general public. Certainly, non-opioid analgesics, such as Tylenol and ibuprofen, are used by Canadians for many forms of pain, including joint pain [40–41], headaches [2], menstrual pain [42], and indeed, mild-to-moderate forms of dental pain and tooth sensitivity [7]. In this regard, the rate of use for these analgesics may simply reflect the ubiquity with which these pain killers are used by those in BC. The slight increase in use of these analgesics by those who have experienced a recent toothache may illustrate the general public’s confidence in these pain killers to provide relief of mild-to-moderate pain [43].

In contrast, almost 15 per cent of those who experienced a toothache in the past month reported taking prescription opioids, such as Demerol, codeine, or morphine–more than twice that of those who did not experience a toothache during the same timeframe (7.1%). There are several plausible explanations for these findings. The first explanation is that some respondents experienced a toothache, and sought appropriate dental treatment for which short-term post-operative opioid analgesics were prescribed as part of their care. This explanation, however, is questionable given the pattern of dental care utilization among respondents. In comparison to those who did not report a dental visit, those who reported a dental visit within the past year were no more likely to have taken Demerol, codeine, or morphine–irrespective of having experienced a toothache or not. Utilization patterns instead are more predictive for the use of painkillers such as aspirin or Tylenol: those who experienced a toothache in the past month and reported a dental visit within the past year were almost twice as likely to have used such analgesics, possibly reflecting the preference of dental professions to recommend these analgesics for patients experiencing various types of acute tooth pain [7, 44].

Another explanation is that those who experience severe or debilitating forms of pain for which opioids are prescribed may simply be more susceptible to experiencing other forms of pain–dental pain included–but in this case, did not take prescription opioids specifically in response to a toothache. Chronic pain has been correlated with a variety of social determinants of health [45–46], suggesting that a person’s pain may be due, at least in part, to factors outside of their control. Indeed, a person’s experience of pain is often exacerbated by the social conditions in which they live: the burden and severity of pain is strongly associated with mental and physical stress at work, socioeconomic status, rurality, employment status, neighbourhood, and education [47]. Likewise, many of these same social and economic factors have been linked to poorer oral health outcomes [48–50], and this may be true for tooth pain, as well [51]. In this regard, chronic pain and dental pain may be related, but not entirely separable using the parameters of this study. However, our incorporation of several comorbidities and chronic conditions which may predispose individuals to being prescribed opioids questions the power of this explanation. Instead, these findings may point to a more direct relationship; individuals who experienced a toothache in the past month used opioid analgesics specifically to relieve their tooth pain, although the prescribed opioid may not have been intended for dental pain originally. To be sure, oral health is influenced by a variety of social and economic factors [48–50], with poorer self-reported and clinical oral health outcomes concentrated among those are more socially and economically marginalized in Canada [52–54]. As well, those from lower socioeconomic positions experience a greater burden of tooth pain: specifically, those with less education and less income report more frequent and more severe tooth pain than their higher socioeconomic counterparts [51, 55].

Our finding of potential income inequalities for prescription opioid use among those who have experienced a toothache in the past month supports this assertion. Before controlling for comorbidities, those from the lowest income bracket were more likely than those from the highest income bracket to report having used prescription opioids, perhaps reflecting a greater burden of disease among those who are poorer. Alternatively, it may point to greater barriers to accessing those dental services necessary to provide pain relief for those with untreated dental needs. These income-related differences for opioid use are consistent with results from a recent study exploring opioid use in the broader BC population [39]. Significantly, though, our study identified income-related differences for opioid use *only* among those who reported a toothache in the past month (that is, no real income effect exists for prescription opioid use in the *absence* of a toothache). Given that those from lower socioeconomic backgrounds disproportionately suffer from untreated dental needs [56–57], our findings suggest that policies designed to improve access to dental services for those who are most socially and economically marginalized may, in part, assuage the need for opioid analgesics among those in BC by either preventing, or directly treating, the cause of pain.

Notably, however, after adjusting for potential confounders–namely, age, sex, mental health indicators, and pain-related comorbidities–the income effect from our unadjusted model disappears. Although these results appear to indicate that the control variables are indeed confounders (i.e. chronic conditions and mental health status are the true predictors of differences in opioid use among those who do and do not experience tooth pain), we hesitate to accept this interpretation for two reasons. The first reason is that chronic pain and poor mental health may not in fact act as confounding variables, but may instead simply be mediators along the pathway leading to the use of prescription opioids. That is, a poor socioeconomic status may influence the distribution of opioid prescriptions in BC, and these pain relievers may be used to relieve any form of pain, be it chronic back pain, tooth pain, or any other type of pain. This may reflect the prescribing habits and implicit biases of physicians and dentists, for instance, or it may be a result of how pain is described or communicated by different social classes. If this is the case, then again, this would support policies designed to improve access to dental services to mitigate the need for prescription analgesics; however, the existing evidence is limited, and both supports [58–59] and refutes [60–61] this theory. The second reason we question the outright validity of the adjusted model is due to a potential violation of the major assumption supporting the use of confounding variables. Specifically, the principle of *ceteris paribus*–“all other things being equal”–is fundamental to the use of control variables [62]. In this case, however, the control variables are not equal; chronic conditions are well-understood to markedly differ across socioeconomic groups with respect to the type of illness, frequency or prevalence of disease, and the severity of the condition [63–66]. In other words, they hold constant something that is not constant in the real world, thereby producing a result that may be an inaccurate depiction of events as they play out in the real world.

Our findings also revealed an income effect for the use of non-opioid analgesics. In contrast to opioids, however, the pattern is reversed: those with less income are considerably *less* likely to have taken pain killers such as aspirin or Tylenol in the past month. This division exists for those who did not report a toothache in the past month, but becomes more pronounced in the presence of a recent toothache. This observation may be suggestive of an unequal distribution in the *severity* of tooth pain; those with less income may experience more severe forms of dental pain that is not alleviated by conventional painkillers such as Tylenol, and accordingly, require prescribed opioids for appropriate pain relief. This explanation again supports an expansion of those dental care programs that are targeted at the poor. Moreover, given that oral health is a product of a wide range of social and economic factors, an “upstream” policy approach that focuses on addressing the social determinants of health is essential.

The *RII* for analgesic use illustrates the magnitude of socioeconomic inequalities in this outcome. Specifically, we found a marked income effect for opioid use within the past month among those who experienced a toothache (*RII*: 2.06; 95% CI: 1.75–2.37). This compares to a statistically non-significant *RII* for those who did not report a toothache during the past month (*RII*: 1.02; 95% CI: 0.87–1.17). Using the latter as a control group, this suggests that opioid prescriptions for those who experience a toothache is roughly twice as high among those from the lowest income brackets compared to those with the highest income. To our knowledge, our study is the first to assess the magnitude of socioeconomic inequalities for the pattern of prescription opioid use to relieve dental pain. On the other hand, our findings support a host of others which have demonstrated (using the *RII)* the existence of socioeconomic inequalities for opioid-related fatalities [29, 67], other forms of pain [68–69], and poorer self-reported and clinical oral health outcomes [36, 70]. This is particularly relevant, given that socioeconomic inequalities are essentially reversed for conventional analgesics. We found that the use of painkillers such as aspirin and Tylenol was considerably higher among those from the highest income households after experiencing a toothache (RII: 0.63; 95% CI: 0.35–0.91) relative to those who did not experience a recent toothache (RII: 0.82; 95% CI: 0.73–0.91). Again, using the latter group as a control, this suggests that conventional analgesic use is almost 25 per cent higher for those with the highest income relative to those with the least income–representing a reversal of the socioeconomic inequalities observed for opioid use. In this regard, adverse socioeconomic conditions appear to influence the need for particular analgesics to relieve dental pain among those living in BC.

Another important consideration is that existing pharmaceutical drug coverage arrangements reinforce these social class differences. Specifically, the BC government finances prescription drug coverage for those who qualify. Generally speaking, these publicly-financed prescription drug plans are tailored to those with less income, the policy rationale being that those with more income are more likely to have employer-sponsored or privately-purchased prescription drug plans, and are more likely to be able to afford out-of-pocket payments for prescription medications. However, analgesics such as Tylenol, naproxen, or ibuprofen, for instance, are classified as non-prescription medications–often referred to as over-the-counter (OTC) medications. Accordingly, OTC medication are not eligible for financial coverage through pharmaceutical insurance plans. As a result, those with less income may be forced to take existing prescription analgesics, intended for the relief of other forms of pain (namely, opioids such as codeine or oxycodone) rather than safer, but more expensive analgesics, such as Tylenol or ibubrofen. In this sense, inequalities for prescription opioid use to relieve mild-to-moderate pain may become even more pronounced between the poor and the rich, for whom costs of non-prescription analgesics are a comparatively smaller financial burden.

Some have recently proposed that pain should be categorized as a social determinant of health, given that it can lead to increased stress levels, workplace hazards, employability, poor nutrition, poverty, and accompanying psychological, mental, and general health problems [71]. Certainly, the existence of such marked income-related differences in the need for particular analgesics may support this idea. If the likelihood to take a prescription narcotic is disproportionately inflated for those with less income, then the risk of adverse drug events–namely abuse and addiction–are necessarily inflated for these people, along with the accompanying sequelae, such as family and relationship challenges, health problems, unemployment, and premature death, for instance [72]. In this respect, “upstream” solutions which attempt to prevent or treat pain in order to mitigate the use of prescription narcotics may help to narrow differences in health between the rich and the poor, and provide social and economic benefits to the broader society [73–74].

Of course, these findings must be considered within the context of the rise of opioid prescriptions in Canada during the past two decades. To be sure, successful pharmaceutical marketing in both Canada and the United States played a critical role in the initial embracing by the medical community to more readily consider opioids for the treatment of chronic non-cancer pain [75]. However, it is increasingly clear that the over-prescription of opioids since then has been a major upstream driver of the opioid abuse epidemic [76]. This rise in opioid over-prescribing by Canadian health professionals has generally been attributed to a lack of consensus among health care providers on whether and how to use opioid analgesics for chronic non-cancer pain [77], and a failure of patients to not use prescription opioid analgesics as directed [78]. Importantly, however, recent guidelines developed by the National Pain Centre in Canada attempt to eliminate this ambiguity by issuing clear directives about when and how to prescribe opioids for chronic pain [79]. These guidelines include: optimizing non-opioid pharmacotherapy and non-pharmacological therapy as first-line treatment for patients; avoiding the use of opioids in patients with an active or prior history of substance abuse; and, stabilizing psychiatric disorders prior to initiating a trial of opioid analgesics, among other recommendations. Nevertheless, the first Canadian guidelines for prescribing opioids in the management of non-cancer chronic pain were released in 2010, and demonstrated only a limited impact on prescribing rates in British Columbia, at least in the short term [39].

It is important to consider the limitations of this study. First, the study was unable to control for several exogenous factors which may act as confounding variables, including: factors that affect pain tolerance; and medications and conditions which create contraindications for use of pain medications [80]. The control group in this study is intended to address this by establishing a baseline group against which the affected group can be compared; that is, the only variable putatively separating each group is the presence or absence of having experienced a toothache within the past month. As well, the study was able to identify and control for several other confounding variables in the regression model, including the age and sex of respondents, potential pain-generating conditions, such as fibromyalgia, arthritis, rheumatism, and back pain, as well as recent injuries or repetitive strain injuries. Moreover, we successfully identified and controlled for several psychosocial variables, including self-reported mental health, life satisfaction, and self-reported daily stress. The second limitation of the study is that its design is cross-sectional in nature, limiting our ability to draw conclusions about causal relationships. Provided with adequate data, a longitudinal study design would better allow for social and economic influences on prescription opioid use to be assessed, while also providing more insight into a causal relationship between the dependent and independent variables. Future research examining this topic may also benefit from using more direct metrics to assess opioid use to alleviate tooth pain, as well as exploring the issue in other Canadian jurisdictions experiencing public health challenges [17, 23] related to prescription opioid use.

## Conclusion

In this study, we identified particular social and economic factors that are associated with using opioid analgesics to potentially alleviate tooth pain among those living in BC. Expanding and improving dental care programs that benefit the poor, and applying policy approaches that focus on addressing the social determinants of health, may help to reduce the use of prescription opioids, recently described by provincial health officers as a “public health emergency.” Such efforts may ultimately help to narrow differences in health between the rich and the poor, providing social and economic benefits to the broader population.
